# Computational and biochemical characterization of two partially overlapping interfaces and multiple weak-affinity K-Ras dimers

**DOI:** 10.1038/srep40109

**Published:** 2017-01-09

**Authors:** Priyanka Prakash, Abdallah Sayyed-Ahmad, Kwang-Jin Cho, Drew M. Dolino, Wei Chen, Hongyang Li, Barry J. Grant, John F. Hancock, Alemayehu A. Gorfe

**Affiliations:** 1 University of Texas Health Science Center at Houston, Department of Integrative Biology and Pharmacology, 6431 Fannin St., Houston, Texas, 77030, USA; 2 University of Michigan Medical School, Department of Computational Medicine and Bioinformatics, Ann Arbor, Michigan, USA

## Abstract

Recent studies found that membrane-bound K-Ras dimers are important for biological function. However, the structure and thermodynamic stability of these complexes remained unknown because they are hard to probe by conventional approaches. Combining data from a wide range of computational and experimental approaches, here we describe the structure, dynamics, energetics and mechanism of assembly of multiple K-Ras dimers. Utilizing a range of techniques for the detection of reactive surfaces, protein-protein docking and molecular simulations, we found that two largely polar and partially overlapping surfaces underlie the formation of multiple K-Ras dimers. For validation we used mutagenesis, electron microscopy and biochemical assays under non-denaturing conditions. We show that partial disruption of a predicted interface through charge reversal mutation of apposed residues reduces oligomerization while introduction of cysteines at these positions enhanced dimerization likely through the formation of an intermolecular disulfide bond. Free energy calculations indicated that K-Ras dimerization involves direct but weak protein-protein interactions in solution, consistent with the notion that dimerization is facilitated by membrane binding. Taken together, our atomically detailed analyses provide unique mechanistic insights into K-Ras dimer formation and membrane organization as well as the conformational fluctuations and equilibrium thermodynamics underlying these processes.

Ras proteins are intracellular guanine tri-phosphate (GTP) hydrolyzing enzymes (GTPases) that mediate signal transduction from the extracellular environment to the nucleus^1^,^2^. Signaling through Ras is achieved via a switch-like off/on conformational change driven by guanine di-phosphate (GDP) and GTP exchange. Malfunction in the switching function of the three human Ras isoforms N-, H- and K-Ras (4 A&B) due to somatic mutations is linked to 15–25% of all human cancers. Up to 85% of these are due to mutations in K-Ras4B (here after K-Ras), and include some of the most lethal cancers such as pancreatic and colorectal cancers^3^. K-Ras interacts with effectors and exchange factors via a conserved catalytic domain comprising the first 166 of 185 residues, and with the plasma membrane (pm) via a farnesylated C-terminus carrying six lysines. The isolated catalytic domain as well as full-length K-Ras can bind effectors and hydrolyze GTP in their monomeric form^4^,^5^. Previous studies thus largely focused on the monomer even though Ras dimerization has been proposed as far back as 1988^6^ and 2000^7^. This changed recently with the finding that dimers of endogenous K-Ras activate the MAPK pathway^8^.

There is evidence that Ras dimers also form in synthetic membranes. Gerwert and colleagues used fluorescence energy transfer (FRET) and Fourier transform infrared (FTIR) spectroscopies plus molecular dynamics (MD) simulation to propose that N-Ras forms dimer in a POPC bilayer^9^. Similarly, using fluorescence correlation spectroscopy (FCS) and other techniques Groves and colleagues proposed dimerization of H-Ras in a supported bilayer^10^ due to oxidative covalent interactions^11^. The evidence for Ras dimerization in solution is mixed. For example, N-Ras was shown to dimerize in bulk solution at concentrations as low as 0.34 μM while K-Ras is a monomer at 2.0 μM^12^. Yet the soluble catalytic domain of K-Ras was reported to dimerize with an apparent dissociation constant (K_d_) of ~1 μM^13^, while a C-terminally cross-linked H-Ras does not^14^. Moreover, it is currently unclear whether Ras oligomerization involves direct protein-protein interactions or is indirectly mediated via membrane association. Some studies supported direct interaction involving the catalytic domain and proposed several possible interfaces^9^,^13^. Direct protein-protein interaction has also been found in other small GTPases such as Arf^15^, Rab^16^ and Sar1^17^. However, dimerization primarily mediated by the polybasic C-terminal membrane anchor has been reported for Rho^18^ and Rsr1^19^. The discrepancies may in part reflect the dependence of dimerization on specific conditions, a notion supported by the enhancement of K-Ras dimerization by the GTP analogue GTPγS^13^. In cells, super-resolution microscopy suggested that the farnesylated lipid anchor is involved in dimerization of K-Ras^8^, but there is no data about the role of the catalytic domain.

We hypothesized that K-Ras dimerization involves direct yet weak protein-protein interactions (PPIs), so that the population of dimers is too small to be observed without the concentrating effect of membrane. Here we show that the catalytic domain of K-Ras is indeed directly involved in the formation of multiple dimers, but the PPIs are so weak that dimers are unlikely to be observed under standard experimental conditions in solution but can be enriched upon membrane binding. We arrived at this conclusion after a comprehensive analysis of data from multiple sources, both experimental and computational. Our modeling combined sequence/structure analyses, protein-protein docking, molecular simulations and free energy calculations. Our experiments included biochemical assays in cells that, guided by the computational data, were specifically designed to probe PPIs. We also show how diverse K-Ras dimers can emerge from two proximal PPIs.

## Results

**Figure 1:**
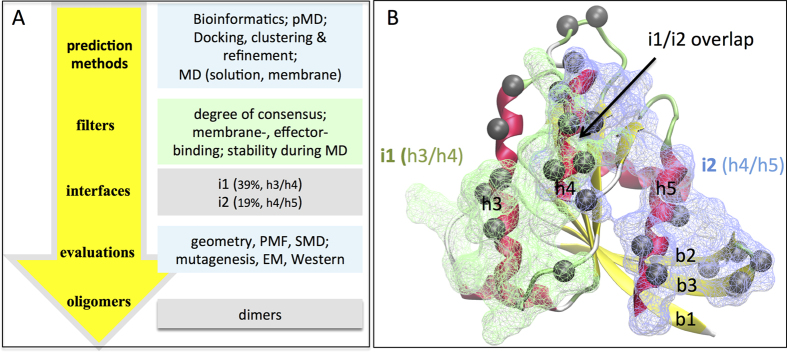
Summary of our approach and key findings. (**A**) Workflow for prediction and validation of K-Ras oligomers/interfaces. MD, pMD, SMD, PMF and EM represent molecular dynamics, probe-based MD, steered MD, potential of mean force and electron microscopy, respectively. (**B**) Location of interfaces i1 (green) and i2 (blue) on the three-dimensional (3D) structure of K-Ras shown in cartoon with α-helices (h) in red and β-strands (b) in yellow. Grey spheres are the C_α_ atom of co-evolving residues with score >2.0 (Supplementary Material). Secondary structure elements extensively discussed in the text are labeled: helices h3, h4 and h5; strands b1, b2 and b3. Notice a remarkable overlap between predictions from protein-protein docking and simulation (wireframes) and sequence co-evolution analysis (spheres). i1 and i2 share residues at h4 and therefore have the potential to form a single continuous interface.

Figure 1 summarizes our overall approach and key findings. This includes the application of a range of complementary computational and experimental methods that collectively highlighted two major oligomerization interfaces.

### Reactive surfaces on K-Ras

A monomer susceptible for oligomerization often has solvent exposed conserved hotspot residues that have a comparatively high propensity for interaction^20^. In previous studies, we probed the surface of K-Ras for its ability to interact with organic molecular fragments using probe-based MD simulations (pMD)^21^,^22^. We found significant probe densities on the surfaces of lobe 2 (residues 87–166) helices 3 and 4 (h3/h4), and turn b2/b3 on lobe 1 (residues 1–86). As these sites did not overlap with known ligand binding sites and were largely solvent exposed, we proposed that they might represent potential PPIs^22^. Consistent with this hypothesis, our sequence based bioinformatic analysis identified co-evolving surface residues localized to h3/h4 and to a lesser extent turn b2/b3 and helix 5 (h5) (Fig. 1B; Table S1). These findings indicate that conserved surface residues in these regions are potentially reactive (Fig. 1B). These surfaces are also known to participate in membrane binding^23^,^24^,^25^ and crystal contacts^26^,^27^. Taken together these observations demonstrate that helices 3–5 and especially h3/h4 are the most potentially reactive surfaces on lobe 2 of K-Ras.

### Two partially overlapping PPIs on K-Ras

Native oligomers are mostly symmetric^28^. Therefore, we first conducted symmetric protein-protein docking of K-Ras with RosettaDock^29^, and obtained a total of ten clusters (see Methods; Figure S1). Of the top six clusters with >5% enrichment, all but one have a helix-helix interface: h3/h4 (C1 & C2), h4/h5 (C3 & C5) and h2/h3 (C4). The interface in cluster C6 involved the switch regions and beta strands. Among the minor clusters, C8 shares similarity to C3 and C5 while the interfaces of C7, C9 and C10 involved switches and loops. Application of a 5% cluster size cutoff and our empirical filters, namely, that the interfaces should not be made up of only loops or bury the effector-binding loop (see Methods), eliminated C6–10 and left us with five clusters. To these clusters we added one minor cluster from an asymmetric docking that resembled C3 (0.1 nm C_α_ RMSD between their representative structures).

**Figure 2:**
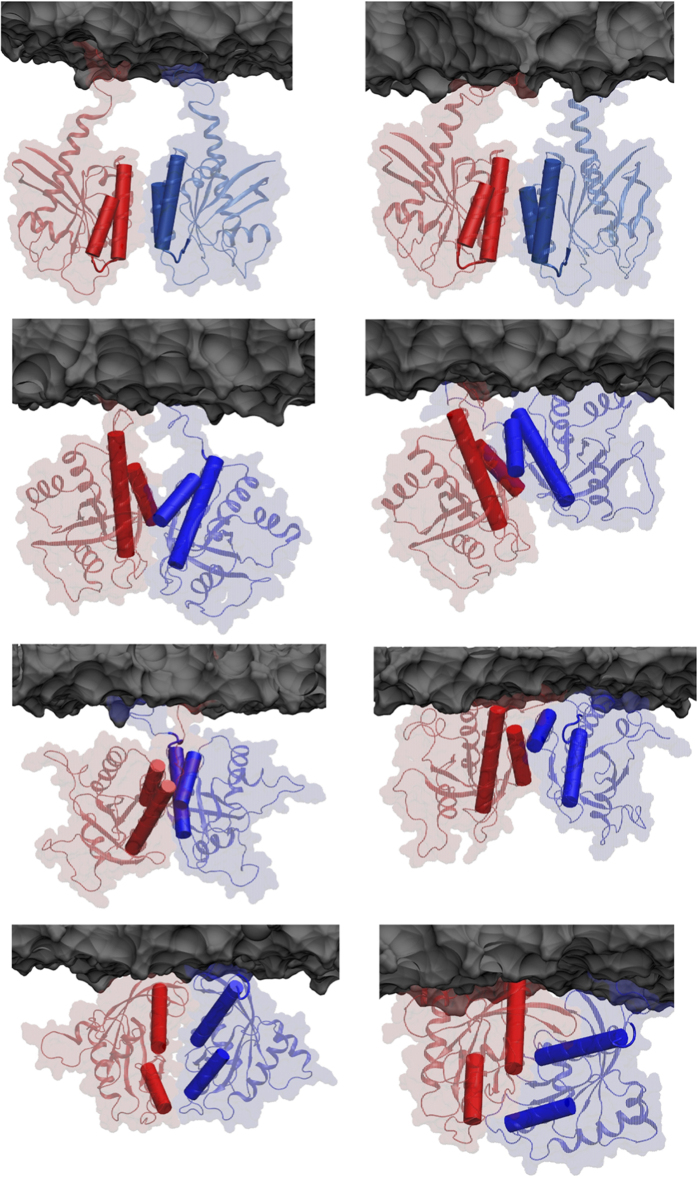
The final four predicted dimers. The top two rows represent dimers with interface i1 (row 1) and interface i2 (row 2) and rows 3 and 4 represent two other variants of i2. The snapshots illustrate complexes after protein-protein docking, filtering and membrane anchoring (left column) and subsequent optimization by MD (right column). Monomers are in red and blue cartoon with background surfaces. Interfacial helices h3/h4 (row 1) and h4/h5 (row 2–4) are highlighted as rods. A portion of the bilayer surface is shown in grey.

A closer look at the predicted binding modes within each of the six clusters showed substantial variation in the relative orientation of apposed helices (compare C1 with C2 and C3 with C5 in Figure S1). We reasoned that MD simulation in membrane might remove strains and optimize interfacial interactions, so that viable interfaces with slightly different initial orientations may converge while high-energy unstable dimers dissociate completely or lose significant interfacial area. Thus, centroids of the six clusters (two h3/h4, three h4/h5 and one h2/h3) were subjected to all-atom MD in a POPC/POPS bilayer. One of the h3/h4 dimers and the h2/h3 dimer dissociated during the simulations (Table S2), suggesting that these interfaces are not viable. One of the h3/h4 and all three of the h4/h5 dimers did not dissociate (Fig. 2; Table S2). Instead, they underwent reorganizations that improved the inter-monomer interactions and increased the buried interfacial surface area (Supplementary Material; Table S2; Figures S3, S4). Dissociation of one of the h3/h4 dimers but not the other highlights the importance of the relative orientation of helices to the PPI, whereas the stability of all three h4/h5 models underscores how various dimers can be built from the same reactive surface.

For further analysis we selected one h3/h4 (referred to as i1 from hereon) and one of the h4/h5 (i2) models. For the latter, we chose the model that exhibited the largest improvement during the simulations in terms of inter-monomer interaction energy and buried surface area (Table S2; Figures S3 and S4). As a result of reorganization during the simulations, both i1 and i2 deviated from their initial perfect symmetry to become quasi-symmetric, which is common in oligomers^30^. The final arrangement of h3 and h4 in i1 is semi-parallel while that of h4 and h5 in i2 is nearly perpendicular with a ~70° angle between the axes of h5. This made i1 similar to the crystal packing observed in the PDB structures 4LV6 and 4LUC and i2 to a proposed dimer model of N-Ras^9^ and the crystal contacts in 5P21^31^. We note that i1 and i2 – though distinct (Fig. 1B)–share a set of common residues at h4 and thus partially overlap.

### K-Ras dimers have weak PPIs

**Figure 3:**
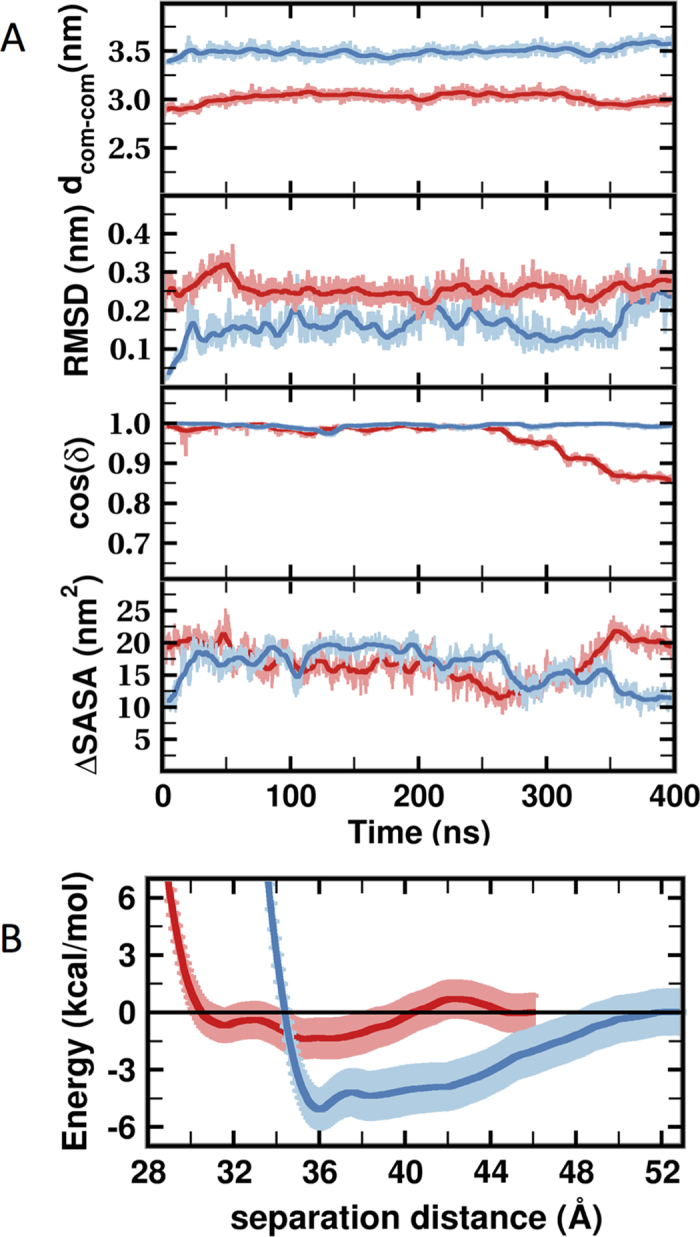
Quantitative characterization of i1 and i2. (**A**) Time evolution of inter-monomer center-of-mass distances (d_COM_), C_α_ root-mean-square deviation (RMSD), relative orientation of monomers (δ), and interfacial solvent accessible surface area (ΔSASA). Data were sampled every 100 ps, with the thick solid line highlighting running average over 10 ns. (**B**) One dimensional (1D) PMF for i1 (blue) and i2 (red) along d_COM_. The minima are assumed to represent the bound states while the reference unbound state is assumed to be at d_COM_ > 4.6 nm. Error bars were obtained from uncertainties in the mean force.

As a further test of viability, we simulated i1 and i2 in water. We did not observe any spontaneous dissociation of either model. For example, the time evolution of the RMSD relative to the initial structure shows no major conformational change (Fig. 3A). The d_COM_ also remained stable at 3.5 ± 0.1 nm and 3.0 ± 0.1 nm for i1 and i2, respectively. Though i2 exhibited a comparatively larger fluctuation in terms of the relative orientation of the monomers (Fig. 3A) the interface remained intact in both simulations. This allowed us to derive ensemble-averaged interfacial features that can be compared with literature data. These include average ΔSASA of 18 ± 2 nm^2^ and 16 ± 3 nm^2^ respectively for i1 and i2, which are within the 19 ± 8 nm^2^ expected range from statistical analysis of known PPIs^32^. Similarly, the number of interfacial HCs (13 ± 3 and 8 ± 3) and HBs (8 ± 2 and 5 ± 2) in i1 and i2 are close to the values (~12 and ~10) expected for stable PPIs^33^. These results suggest that i1 is somewhat more stable than i2, consistent with the multiplicity of i2 dimers discussed earlier. We draw the same conclusion from the PMFs in Fig. 3B, showing that i1 has a relatively deep free energy minimum at d_COM_ = 3.6 nm, a comparable distance to the 4.6 ± 0.6 nm measured by FRET for N-Ras^9^. In contrast, i2 has multiple shallow minima at ~3.6 nm and 3.2 nm. The appearance of minima in the PMFs upon inter-monomer contact provides a thermodynamic basis for the formation of dimers via the two interfaces. At the same time, the shallowness of the minima indicates small relative free energy changes and therefore marginal stability. Moreover, while both are held together by very weak forces i1 is significantly more stable than i2. This difference is fairly reliable considering the relatively small errors in the PMFs and the fact that i2 was more labile during the simulations.

### Key interfacial interactions stabilizing the putative K-Ras dimers

**Figure 4:**
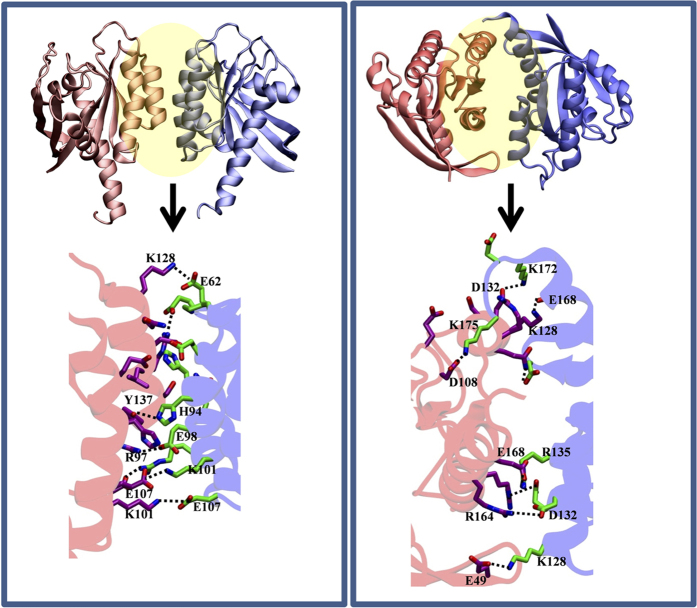
Interfacial interactions. (Top) The global structure of the i1 and i2 dimers colored as in Fig. 2. (Bottom) Close-up views of inter-monomer side chain interactions (some of them labeled) with carbons colored purple (monomer 1) and green (monomer 2), oxygen red and nitrogen blue.

We analyzed interfacial contacts in the MD trajectories and conducted computational alanine scanning mutagenesis to reveal key residues contributing to inter-monomer interactions (see Supplementary Material). Figure 4 (see also Figure S5) highlights selected interfacial residues, particularly those involved in inter-monomer electrostatic interactions. For i1, these include residues 94, 95, 97–99, 101, 102, 107, 128, 133, and 135–137. In particular, salt bridges E98-R97 and K101-E107 as well as a π-stacking interaction between side chains of H94/H95 and Y137 are critical. Similarly, i2 is stabilized by E49-K128 and R135-E168 and a number of other polar interactions (Fig. 4).

### Experimental validation

**Figure 5:**
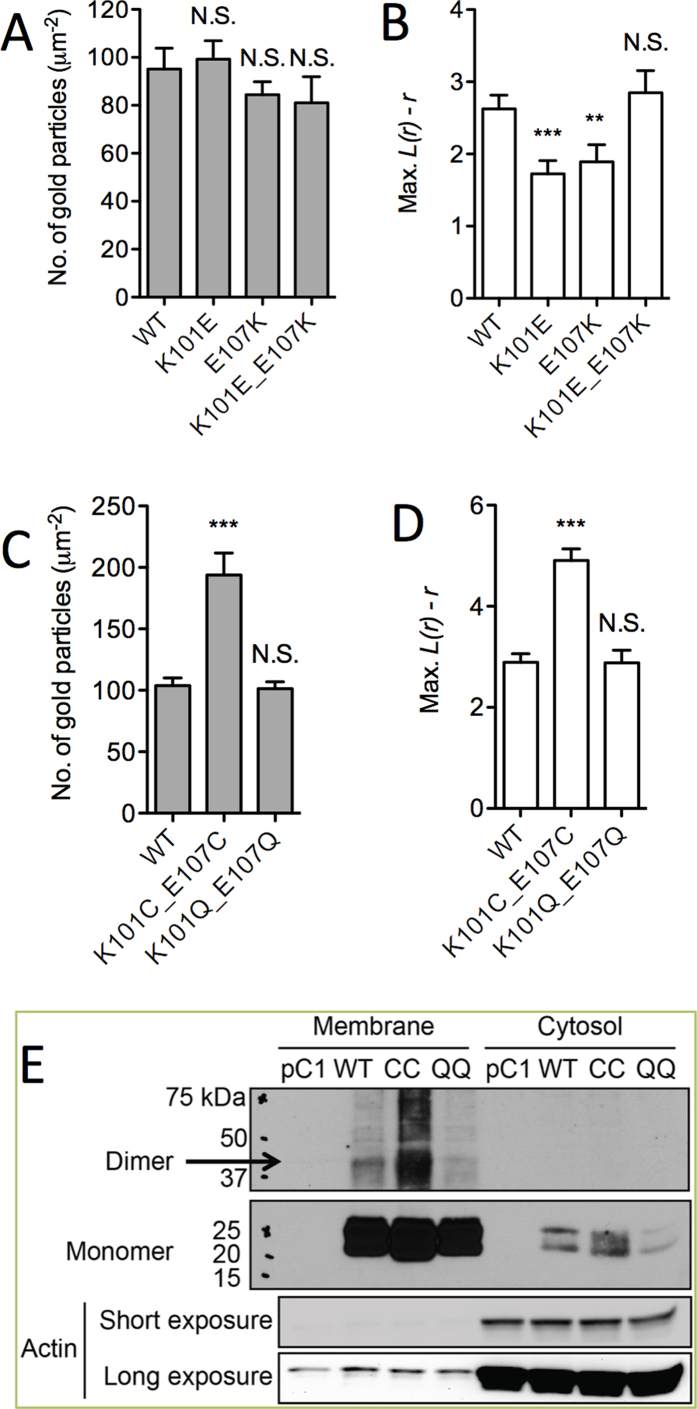
EM and Western analysis of K-Ras dimers/oligomers. (**A**–**D**) Plasma membrane (pm) sheets from BHK cells expressing mGFP-K-RasG12V mutants were labeled with anti-GFP conjugated gold and visualized by EM. (**A**,**C**) Mean number of gold particles/μm^2^ (±S.E.M., n ≥ 20) with significant differences assessed by one-way ANOVA tests (***p < 0.001, N.S. = Not significant); (**B**,**D**) peak values of the weighted mean K-function *L(r)-r* curves (max. *L(r)-r*) are shown) with significant differences evaluated by bootstrap tests (**p < 0.01, ***p < 0.001). (**E**) BHK cells transiently expressing HA-tagged K-RasG12V mutants or an empty vector (pC1) were harvested with isotonic lysis buffer without DTT. Cell lysates fractionated into membrane and cytosolic fractions were denatured in Laemmli sample buffer without DTT and immunoblotted with an anti-HA antibody. Representative blots from three independent experiments are shown. An actin blot is shown as loading control.

Our computational analysis identified K101-E107 and E107- K101, among others (Figs 4 and S5), as key inter-monomer salt bridges that stabilize the largely polar interface of an i1-mediated dimer. We disrupted these ionic interactions by replacing one of the partners with an amino acid of the opposite charge and measured the extent of K-Ras clustering including dimerization. To this end, intact pm sheets from BHK cells expressing mGFP-tagged K-RasG12V (WT), K101E and E107K mutants were labeled with anti-GFP antibody conjugated with 4.5 nm gold particles, imaged with EM, and their spatial organization analyzed by Ripley’s K-function. The results summarized in Fig. 5 show that K101E and E107K each significantly reduced clustering without affecting membrane binding (Fig. 5A and B). Conversely, clustering was unaffected in the double mutant K101E/E107K, where the apposed charges are simply swapped (Fig. 5A and B). These data suggest that K101 and E107 are involved in K-Ras clustering via an ionic interaction. We tested this further by generating a K101C/E107C (or CC) double mutant. The idea was that if indeed these two residues are facing each other in a self-assembled oligomer then cysteine side chains at these positions might form a disulfide bond that stabilizes the dimer. Indeed, our EM data show that CC significantly enhanced K-Ras clustering and membrane retention, whereas K101Q/E107Q (QQ), which cannot form a disulfide bond but can still form a hydrogen bond, did not affect clustering or membrane binding (Fig. 5C and D). The double cysteine mutation dramatically increased the fraction of dimers and higher oligomers relative to monomers (Figure S6). Together, these observations suggest that inter-monomer ion pairs between residues 101 and 107 contribute to K-Ras oligomerization and membrane binding.

We further examined K-Ras dimerization directly by immunoblotting protein samples from BHK cells transiently expressing HA-tagged WT, CC or QQ. Cells harvested in an isotonic buffer were fractionated into membrane and cytosolic fractions and protein samples resolved on a non-denaturing gel before immunoblotting with an anti-HA antibody. Under these conditions, we detected a band at ~40 kDa in the membrane fraction of cells overexpressing WT K-Ras, which is an expected MW for a K-Ras dimer (Fig. 5E). This band was much more pronounced in cells expressing CC, but not QQ. These data show that K-Ras forms a dimer(s) and introducing Cys residues in the interface at positions 101 and 107 stabilize the dimer. Taken together with the EM analysis, our experimental data confirms our prediction that K101 and E107 are involved in K-Ras dimerization.

## Discussion

Functional K-Ras dimers have been detected in cells by super-resolution microscopy and biochemical methods^7^,^8^. However, many questions remain unanswered, including (a) if, or how, dimerization facilitates effector binding, (b) if there exist multiple unique dimers with variable potential to interact with partner proteins, membrane or ligands, and (c) whether dimerization prevents random aggregation from increased local concentration upon membrane binding. Addressing these questions requires an atomic-level characterization of the interface(s), structure(s) and energetics of dimers. The challenge is that Ras dimers are not readily observed in solution likely due to marginal stability whereas in cells experimental techniques lack the resolution to visualize the interactions underlying dimers. Previous attempts at modeling Ras dimers, based primarily on analysis of crystal packaging, suggested h5 plus b2/b3 turn^9^ and “beta” or “helical” regions^13^ as being involved in dimerization. However, a full picture of the PPI(s) and the thermodynamics of Ras dimerization remained undetermined. This reflects the challenges of studying Ras oligomers by experiments or their prediction from first principles. Taking these into account, we first postulated that K-Ras self-assembly: (i) occurs on membranes; (ii) is of weak affinity (i.e., low oligomer populations); (iii) involves reactive surface patches on the catalytic domain; (iv) obeys basic principles of assembly including symmetry^34^, buried SASA, and numbers of hydrogen bonds and vdW contacts. Our integrated approach combining diverse techniques ranging from sequence analysis and molecular simulations to cell biology allowed us to derive consensus predictions and validations of K-Ras dimers.

Co-evolution of surface residues in a homo-oligomer can predict roles in inter-monomer contact^35^. Our search for co-evolving residues on the surface of Ras proteins identified hotspots on h3, h4 and to a lesser extent on h5 and turn b2/b3 (Fig. 1B; Table S1). Previously, we found that these surfaces interact with molecular probes or bilayer lipids^23^,^24^,^25^. Combining these observations, we hypothesized that surface residues on h3-5 or b2/b3 may stabilize K-Ras homo-oligomers. Subsequent protein-protein docking followed by cluster analysis and refinement yielded 10 dimer models. Four of these did not satisfy our empirical viability tests (see Methods) but the interfaces in the remaining six involved residues from one or more of h3-5 (Figure S1). That three independent approaches implicate the same regions as interfaces encouraged us to further examine the six dimer models using MD, a critical component of our toolset for testing stability and model refinement. The simulations were conducted first in bilayer and then in water. Two models dissociated during simulation in bilayer and were discarded. In the other four, the interface improved in terms of buried surface area and/or energy (Table S2; Figure S3), accompanied by formation of new or enhancement of existing interactions (Figure S4). Two structures from the last snapshot of the simulations with the most improved interfaces, i1 and i2, were selected as starting points for an additional simulation in water. Both of these simulations were stable with no dissociation of dimers. Therefore, ensemble-averaged properties of the interfaces in these trajectories were compared with literature data (Figs 3,4 and S4). We found that the physicochemical features of i1 and i2 are broadly within the range of bona fide dimers (see Results). Then, combining data from the simulations in bilayer and in water, alanine scanning and SMD, we compiled a list of interfacial residues contributing to dimer formation, and selected a few of those for experimental testing.

**Figure 6:**
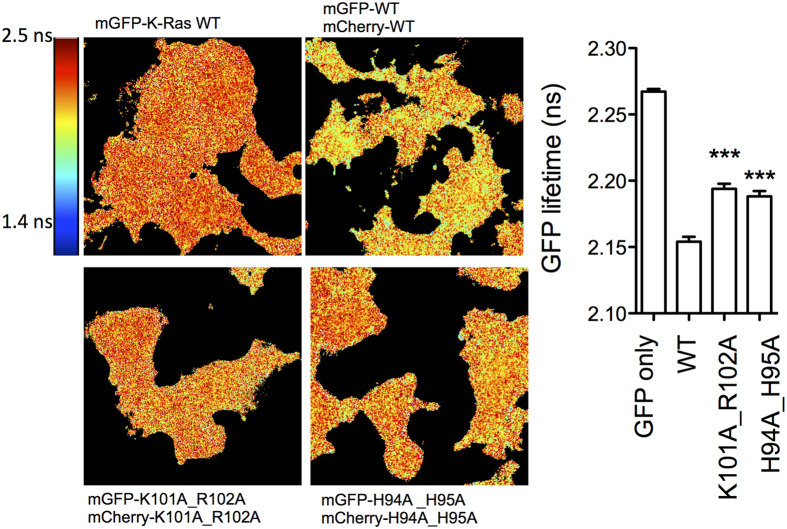
BHK cells were transfected with indicated K-Ras mutants. Cells were fixed with 4% paraformaldehyde and the fluorescence lifetime of GFP was measured using a FLIM microscope. The graph to the right shows mean fluorescence lifetime of GFP +/− SEM. Significant differences between WT (G12V) and mutants replaced with alanine were assessed using one-way ANOVA tests (***p < 0.001).

We chose the comparatively more stable i1 (see below) for validation by biochemical analysis in BHK cells. While studies in purified K-Ras might be more direct, the weak PPIs suggest that the dimers would be too unstable to be captured in solution. In cells, charge reversal mutations at positions 101 and 107 (K101E and E107K) reduced clustering and the fraction of dimers while the double mutant K101E/E107K did not (Figs 5 and S6). Furthermore, introducing cysteine at the putatively apposed 101 and 107 (K101C/E107C or CC) dramatically enhanced clustering, membrane binding and dimer/monomer ratio (Figs 5 and S6). For a more direct illustration of both dimerization and the role of the i1 interface we immunoblotted the membrane fraction of WT and CC K-Ras under a non-reducing condition. Figure 5E shows a ~40 kDa dimer band for both WT and CC, with the CC band being substantially more prominent. Additional bands of higher molecular weight suggest the potential existence of larger oligomers as well. One may argue that introduction of charge repulsion at two sites in a somewhat extended interface might not directly assess the contribution of the ion pairs to affinity. In addition, formation of a disulfide bond in the reducing environment of the cytosol would require the existence of an extended interface that is sufficiently shielded from solvent. We therefore tested if other predicted pairwise interactions in the i1 interface also affect K-Ras clustering. To this end, we mutated two pairs of residues to Ala (H94A/H95A and K101A/R102A), and measured clustering using fluorescence lifetime imaging microcopy (FLIM). Note that H94 and H95 are located at the center of the interface on h3 while the loop 7 residues K101 and R102 are peripheral (Fig. 4). Figure 6 shows that the GFP lifetime of WT K-Ras is much smaller than the GFP control, indicating self-interaction. This interaction is significantly reduced by both H94A/H95A and K101A/R102A mutations. These same mutations also reduced clustering by about 25% when assessed by EM. Taken together these results not only show that K-Ras dimerizes in cells but also establish the critical role of inter-monomer ion pairs and other pairwise interactions throughout the i1 interface.

The next key question was whether i1 and i2 are thermodynamically stable. The calculated ΔSASA and numbers of HBs and HCs (Figs 3 and 4) suggest that both might be stable but that i1 is somewhat more stable than i2. This is consistent with the single i1 and multiple i2 dimers predicted by Rosetta/MD (Figs 2 and S3; Table S2). To probe this more directly, we used our PMFs (Fig. 3B) where i1 exhibits a single relatively deep minimum at d_COM_ = 3.6 nm, in contrast to the multiple shallow minima of i2 at d_COM_ = 3.6 nm and 3.2 nm that are separated by thermally accessible barriers. This implies that interface i1 is modestly stable whereas i2 is malleable and can mediate multiple semi-stable dimers interconverting with one another. For a more direct comparison, we calculated the equilibrium dissociation constants (K_d_) from the PMFs as described in Supplementary Material, assuming that the minima at 3.6 nm represent the bound and the flat profiles at large d_COM_ the reference monomeric state. K_d_ is related to the binding free energy change (ΔG_bind_) and the on and off rate constants k_on_ and k_off_ through K_d_ = exp(−ΔG_bind_/RT) = k_off_/k_on_, where R is the gas constant and T = 300 K is temperature in Kelvin. We obtained K_d_ ≈ 5 mM for i1, which is equivalent to ΔG_bind_ ≈ −3.1 kcal/mol, and K_d_ ≈ 107 mM or ΔG_bind_ ≈ −1.3 kcal/mol for i2. These values show that i1 is ~20-fold stronger than i2, and both have a very weak PPI characterized by a fast off and/or a slow on rate. This agrees with the fact that K-Ras dimer has not been readily observed in solution and suggests weak complexes even when membrane associated. This makes sense from a functional standpoint because K-Ras oligomers should easily disassemble in response to changes in environmental stimuli; transient signaling complexes are common in signal transduction pathways^36^.

## Experimental Procedures

A summary of our integrated computational and experimental approach is provided in Fig. 1A. Below we briefly describe each technique with an emphasis on how they were employed to study Ras dimerization. A more detailed description is provided in Supplementary Material.

### Sequence co-evolution analysis

Co-evolution of surface residues can provide valuable insights into their potential role in oligomerization^37^. Analysis of potentially correlated evolutionary residues involved the use of HMMER (http://hmmer.org), EVcoupling v2.0 (http://evfold.org/evfold-web/citation.do) and Bio3D v2.2^38^,^39^ as described in Supplementary Material.

### Probe binding analysis

Data from previous pMD simulations were used to identify potentially reactive surfaces^21^,^22^, where K-Ras was simulated in the presence of a small organic molecule, isopropanol, both in water and membrane environments. The trajectories were used to examine sites where the probe molecules consistently bind, and solvent exposed flat surface patches with high probe-binding propensity were defined as reactive surfaces.

### Protein-protein docking and filtering

Docking was performed with RosettaDock^29^ using the default two-step procedure: an initial low-resolution step followed by a high-resolution second step. See Supplementary Material for a detailed description of our docking, clustering and refinement protocols. Here we add that, in addition to energy evaluations and cluster analysis, we used three empirical filters in our model selection: (i) the PPI contains co-evolving residues or involves pMD-predicted reactive surfaces, and is not made up solely of flexible loops; (ii) the effector-binding loop of each protomer is solvent exposed and available for effector binding; and (iii) the lipid anchor of each protomer is able to insert into a membrane.

### Molecular dynamics simulation

Two sets of classical MD simulations were conducted to test the stability of predicted models as well as for model refinement. These included simulations of six initial models bound to a POPC/POPS bilayer followed by simulations in water for two of the most stable dimer models. Full details of the system setup and simulations are provided in Supplementary Material.

### Characterization of interfaces

A number of interfacial features were monitored including: (i) solvent accessible surface area (SASA) buried at the interface (ΔSASA), defined as the total SASA (calculated using a 0.14 nm probe radius) of the two monomers minus SASA of the dimer; (ii) number of inter-monomer HBs, defined by a 0.3 nm donor-acceptor distance and a 20° donor-hydrogen-acceptor angle cutoff; (iii) number of HCs, evaluated as the number of carbon atoms in one monomer within 0.38 nm of any carbon atom on the other monomer; (iv) inter-monomer center-of-mass distance (d_COM_); and (v) relative orientation of monomers (Supplementary Material).

### Binding free energy calculation

The change in the free energy of dimerization (ΔG_bind_) can be estimated from a PMF calculated along a suitable reaction coordinate^40^, or similar other techniques (e.g. ref. 41). We calculated PMFs of i1 and i2 dimers along d_COM_ and derived K_d_ and ΔG_bind_ as described in Supplementary Material and in ref. 42.

### EM and spatial mapping

Gold particle-conjugated anti-GFP antibody-labeled, mGFP-tagged K-Ras mutants from BHK cells were imaged using transmission electron microscopy^43^,^44^ and analyzed by Ripley’s K-function (Supplementary Material).

### FLIM measurements

BHK cells were transfected with mGFP- and mCherry-tagged K-Ras mutants and fixed with 4% paraformaldehyde. The fluorescence lifetime of GFP was measured using a FLIM microscope.

### Western blotting

Cells were washed in cold phosphate-buffered saline (PBS) and lysed in isotonic buffer containing 10 mM TrisCl (pH 7.5), 25 mM NaF, 5 mM MgCl_2_, 1 mM EGTA, 100μM NaVO_4_, plus protease inhibitors. SDS-PAGE and immunoblotting with anti-HA (#2999, Cell Signaling Technology) and anti-β-Actin (A1978, Sigma Aldrich) antibodies were performed using 20μg of each lysate. Signal was detected by enhanced chemiluminescence (Thermo Fisher Scientific) and imaged using a FluorChemQ (Alpha Inotech).

## Additional Information

**How to cite this article**: Prakash, P. *et al*. Computational and biochemical characterization of two partially overlapping interfaces and multiple weak-affinity K-Ras dimers. *Sci. Rep.* **7**, 40109; doi: 10.1038/srep40109 (2017).

**Publisher's note:** Springer Nature remains neutral with regard to jurisdictional claims in published maps and institutional affiliations.

## Supplementary Material

Supplementary Information
