# Supplementary material for: Computational and biochemical characterization of two partially overlapping interfaces and multiple weak-affinity K-Ras dimers
Source: Sci Rep. 2017 Jan 9;7:40109. doi: 10.1038/srep40109 (PMC5220301; doi:10.1038/srep40109)
Supplement: Supplementary Information [file srep40109-s1.pdf]

*Supplementary Material*

# Computational and biochemical characterization of two partially overlapping interfaces and multiple weak-affinity K-Ras dimers

Priyanka Prakash[¶1], Abdallah Sayyed-Ahmad[¶1], Kwang-Jin Cho[¶1], Drew M Dolino[1], Wei Chen[1], Hongyang Li[2], Barry J Grant[2], John F Hancock[1], Alemayehu A Gorfe*[1],

[1] University of Texas Health Science Center at Houston, Department of Integrative Biology and Pharmacology, 6431 Fannin St., Houston, Texas 77030

[2] University of Michigan Medical School, Department of Computational Medicine and Bioinformatics, Ann Arbor, Michigan.

*Corresponding authors: Alemayehu A Gorfe (alemayehu.g.abebe@uth.tmc.edu) or John F Hancock (john.f.hancock@uth.tmc.edu)

[¶]*These authors contributed equally to this work.*

**Sequence co-evolution analysis**

To identify potential correlated evolutionary changes we aligned 1524 K-, H- and N-Ras subfamily sequences from the SWISS-PROT/TrEMBL database (http://www.ebi.ac.uk/uniprot date; 11/9/2015). These sequences were identified via a hidden Markov model (HMM) built from a structure based sequence alignment of available Ras crystallographic structures [1]. HMMER v3.1 was used for HMM construction and sequence alignment (http://hmmer.org). The resulting 10,176 sequences were filtered to remove short fragment sequences and clustered to reveal K-, H- and N-Ras subfamilies. Evolutionary coupling analysis was performed on this alignment with EVcoupling v2.0 (http://evfold.org/evfold-web/citation.do). Analysis was restricted to couplings between solvent exposed positions as determined from the ensemble of available Ras crystallographic structures. Bio3D v2.2 [2,3] was used for structure based sequence alignment, alignment filtering and solvent exposure determination. The results are summarized in Table S1.

**Table S1:** Co-evolving residues in Ras proteins*.

| Rank | Residue | Secondary structure | Co-evolution strength | Solvent accessible surface area (Å$^2$) |
|---|---|---|---|---|
| 1 | D132 | h4 | 9.3 | 108 |
| 2 | H94 | h3 | 8.9 | 98 |
| 3 | Q129 | h4 | 7.7 | 87 |
| 4 | D108 | L7 | 7.5 | 105 |
| 5 | S122 | L8 | 7 | 74 |
| 6 | G138 | h4 | 6.3 | 65 |
| 7 | R135 | h4 | 5.5 | 204 |
| 8 | H95 | h3 | 5.2 | 120 |
| 9 | K165 | h5 | 5.2 | 176 |
| 10 | P121 | L8 | 5.1 | 105 |
| 11 | E162 | h5 | 5 | 71 |
| 12 | G48 | L3 | 5 | 68 |
| 13 | E49 | b2 | 4.9 | 60 |
| 14 | E98 | h3 | 4.8 | 74 |
| 15 | R41 | b2 | 4.7 | 139 |
| 16 | T87 | h3 | 4 | 58 |
| 17 | Q43 | b2 | 3.9 | 111 |
| 18 | V45 | b2 | 3.6 | 88 |
| 19 | T50 | b3 | 3.6 | 69 |
| 20 | E91 | h3 | 3.6 | 118 |
| 21 | D126 | h4 | 3.5 | 118 |
| 22 | Y157 | h5 | 3.3 | 53 |
| 23 | Q131 | h4 | 3.2 | 91 |
| 24 | S136 | h4 | 2.7 | 89 |

* Color code: blue (helix h3), yellow (h4), red (h5), grey (β-strands b2, b3), and white (loops L3, L7, L8).

**Protein-protein docking**

The RosettaDock [4] module of the Rosetta program v3.4 was used to conduct symmetric and asymmetric protein-protein docking. We used full-length K-Ras derived from a previous

simulation [5]for docking. Since there is no crystal structure of a Ras dimer for determining the relative orientation of monomers, global docking was conducted with randomized initial poses. Docking was done in two steps using default parameters. The first was a low-resolution step where each residue was represented by a single reaction center, and entailed 500 Monte Carlo (MC) steps of rigid-body rotation and translation. The next refinement step used an all-atom representation of residues and 50 MC steps, in which positions of the monomers were perturbed in random directions and orientations to minimize the energy and optimize side chain conformations [4,6]. The final output consisted of $10^5$ decoys. The top 5% of the lowest-energy decoys (6189 from symmetric and 5000 from asymmetric docking, respectively) were clustered using Calibur [7]. Unless stated otherwise, we selected clusters representing at least 5% of the total number of low-energy decoys for further analysis.

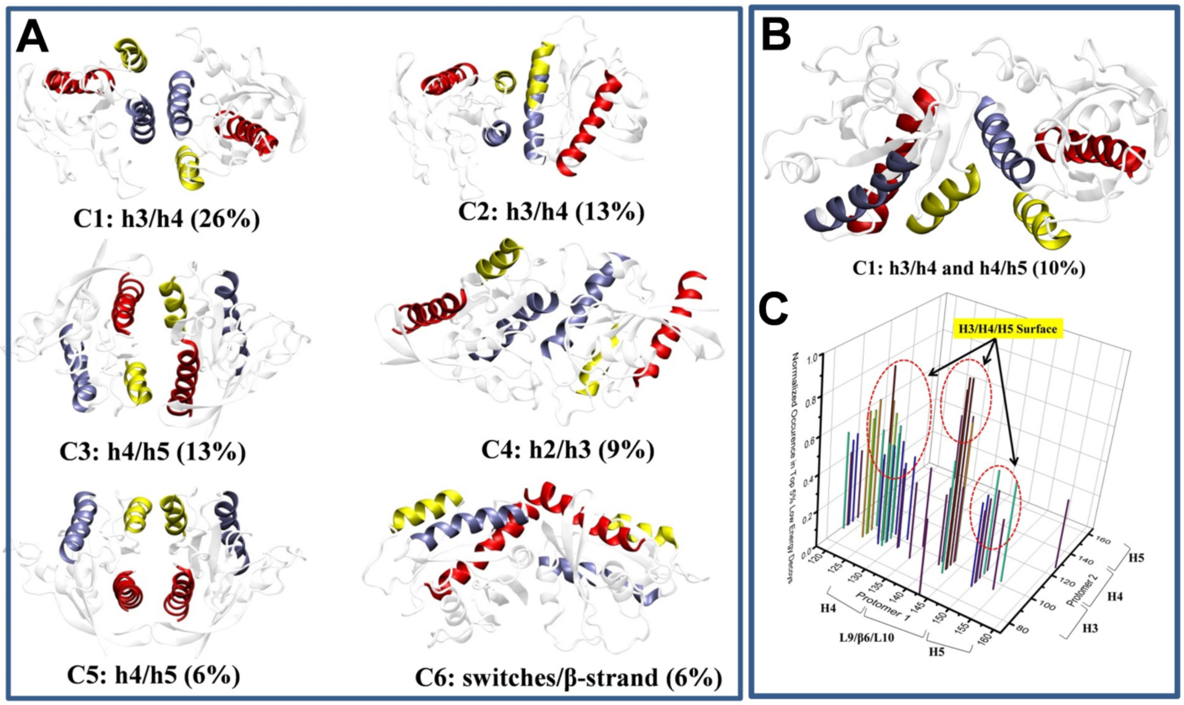


***Figure S1:*** *(A) Representative structures of the top six clusters (>5% occupancy) from symmetric docking with Rosetta. (B-C) Results from asymmetric docking: a representative structure (B) and normalized frequency of pairwise inter-monomer interactions in the top 5% of the low-energy decoys (C). Helix h3 is shown in ice blue, h4 in yellow and h5 in red; the rest of the protein is in white cartoon.*

**Selection and refinement of predicted dimer models**

The top 10 clusters from the symmetric docking accounted for ~86% of the total low-energy decoys, and six clusters contained >5% of the lowest-energy decoys (Figure S1A). In five of these, the dimer interface involved helices h3/h4 (C1 & C2), h4/h5 (C3 & C5) and h2/h3 (C4).

The interface of cluster C6 involves the switch regions and was not considered further (see main text). In the case of the asymmetric docking, the top 10 clusters accounted for ~20% of the 5000 low-energy decoys. Moreover, each of the top three clusters contained only ~3% of the decoys. However, in all three (together representing about 10%), h3/h4 of one protomer interacts with h4/h5 of another, similar to that illustrated in Figure S1B. Since only ~20% of the sample was accounted for by clustering and no cluster contained >5% of the sample, we checked the significance of the asymmetric h3/h4-h4/h5 interactions by calculating the frequency of pairwise inter-monomer residue-residue interactions in all of the 5000 low-energy decoys. Interaction was defined to exist if any non-hydrogen atom of residue i in monomer 1 is within 0.4 nm of any non-hydrogen atom of residue *j* in monomer 2. We found that interaction of residues on h3/h4 with those on h4/h5 is a dominant feature of the ensemble (Figure S1C). These asymmetric h3/h4-h4/h5 interactions appear to be stabilizing higher-order K-Ras oligomers than dimers and therefore not discussed further. However, a minor cluster (~1%) from the asymmetric docking was found to be remarkably similar to the C3 cluster of our symmetric dimer models, with just a small difference in the angle between the interfacial helices. Therefore, we made an exception to our 5% cutoff rule and added this particular cluster to the five symmetric clusters selected for further analysis. The greater emphasis on the symmetric models was because symmetric (or quasi-symmetric) interactions are dominant in oligomers [8].

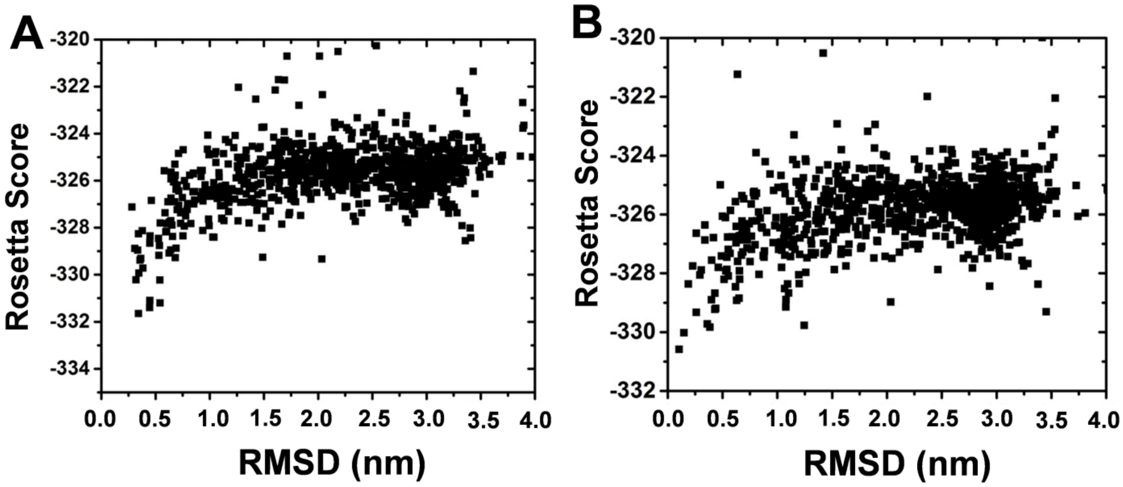


***Figure S2:*** *Scatter plots of Rosetta energy score against RMSD showing funnel-like distributions highlighting the goodness of the predicted h3/h4 (A) and h4/h5 (B) models.*

A representative structure (the cluster center) of each of the six clusters described above was subjected to multiple rounds of perturbation and refinement procedures, which involved a series of rotations and translations followed by energy evaluations [4]. In this way, we generated 1000

dimer structures for each model and plotted their energy score against their root mean square deviation (RMSD) from the initial structure (Figure S2; only two representative plots are shown). The plots exhibit energy funnels [4], indicating that our initial dimer models are the most stable and can be considered native-like. We used these models for further analysis.

**Molecular dynamics simulations**

Two sets of MD simulations were performed: (i) Initial models were attached to a pre-equilibrated bilayer of 320 POPC (1-palmitoyl-2-oleoyl-sn-glycero-3-phosphocholine) and 96 POPS (1-palmitoyl-2-oleoyl-sn-glycero-3-phosphoserine) lipids [5]. After adding water and ions as needed, the resulting ~230,000 atom systems were energy minimized for 2000 steps with lipid and protein heavy atoms fixed, and equilibrated for 200ps with lipid phosphate and protein heavy atoms harmonically restrained with a force constant k = 0.04kcal/mol/nm$^2$, followed by four 100ps runs with k scaled by 0.75, 0.50, 0.25 and 0. The subsequent production phase lasted ~200ns. (ii) Two of the most stable dimer models from (i) were simulated in water for a stringent test of their viability. To keep the overall architecture of the models in their bilayer-bound form while avoiding bias from pre-organized interfacial interactions, we aligned the backbone of the catalytic domain crystal structure 4DSO to each protomer. The resulting 4DSO-based models were re-solvated yielding 100,235 and 83,607 atom systems. Each was then energy minimized for 5000 steps with $C_\alpha$ atoms restrained (k = 0.1kcal/mol/nm$^2$), and equilibrated in five 1ns steps with k progressively decreasing to zero, and then run for 400ns.

For all simulations we used NAMD2.9 [9], CHARMM27 [10] and CHARMM36 [11] force fields for proteins and lipids, respectively, the TIP3P water model, and $Na^+$ and $Cl^-$ ions at a 0.15M ionic strength. Simulation box sizes varied with system size but in each case there was a 1.2-1.4nm space between the edges of the box and solute atom. The isothermal-isobaric (NPT) ensemble at 310K (Langevin dynamics with damping coefficient of 10ps$^{-1}$) and 1.0atm (Nose-Hoover Langevin piston method with piston period 200fs and decay time 100fs) were used. Short-range van der Waals (vdW) interactions were switched off between 1.0nm and 1.2nm and a 1.4nm cutoff was used for non-bonded list updates. The Particle mesh Ewald method was used for calculating electrostatic interactions [12]. Except in the membrane systems where 1fs was used at the equilibration phase, a 2fs time step was used with SHAKE applied to bonds involving hydrogen [13].

**Table S2:** Changes in solvent accessible surface area (ΔSASA, $nm^2$) and non-bonded energy (E, kcal/mol) during MD simulations of K-Ras dimer models in bilayer.

| Model | Interface | $\Delta SASA_{Start}$ | $\Delta SASA_{End}$ | $\Delta\Delta SASA_{End\text{-}Start}$ | $E_{Start}$ | $E_{End}$ | $\Delta E_{End\text{-}Start}$ |
|---|---|---|---|---|---|---|---|
| 1 | h3/h4 | 7.1 | 12.0 | 4.9 | -213 | -299 | -86 |
| 2 | h3/h4 | 10.3 | 0.0 | -10.3 | -166 | -9 | 155 |
| 3 | h4/h5 | 8.5 | 15.6 | 7.1 | -193 | -293 | -100 |
| 4 | h4/h5 | 11.6 | 10.4 | -1.2 | -247 | -474 | -227 |
| 5 | h4/h5 | 12.0 | 18.8 | 6.8 | -504 | -465 | 39 |
| 6 | h2/h3 | 13.3 | 4.8 | -8.5 | -264 | -201 | -63 |

We used the structures described above (two h3/h4, three h4/h5 and one h2/h3) as starting points for the MD simulation in a heterogeneous lipid bilayer conducted as described above. Table 2 summarizes the key results from the simulations, including changes in interfacial solvent accessible surface area (ΔΔSASA) and inter-monomer non-bonded interaction energy (ΔE). We also show in Figure S3 the time evolution of the non-bonded (electrostatics plus vdW) interactions between the monomers. One can see that two dimers (h2/h3 and one of the h3/h4) dissociated quickly (yellow shades in Table S2), whereupon ΔΔSASA became negative (i.e., ΔSASA at the end of the simulation became zero or significantly diminished) while ΔE became positive (E increased); these models were deemed unstable. In one of the h4/h5 models there was only a small change in ΔSASA but a large change in E while in another E was unchanged and ΔSASA significantly increased (white shade in Table S2); these models were deemed stable. There were large favorable changes in both ΔSASA (increased) and E (decreased) in one each of the h4/h5 and h3/h4 models (green shades), indicating substantial reorganization leading to improved interactions at the interface (green shades in Table S2); these models were deemed best. Taken together, these results indicate that four of our six models are potentially viable. For further analysis, we selected the two best models that exhibited the largest favorable changes in both ΔSASA and E as representatives of h3/h4 and h4/h5 interfaces, and refer to them as interface 1 (i1) and interface 2 (i2).

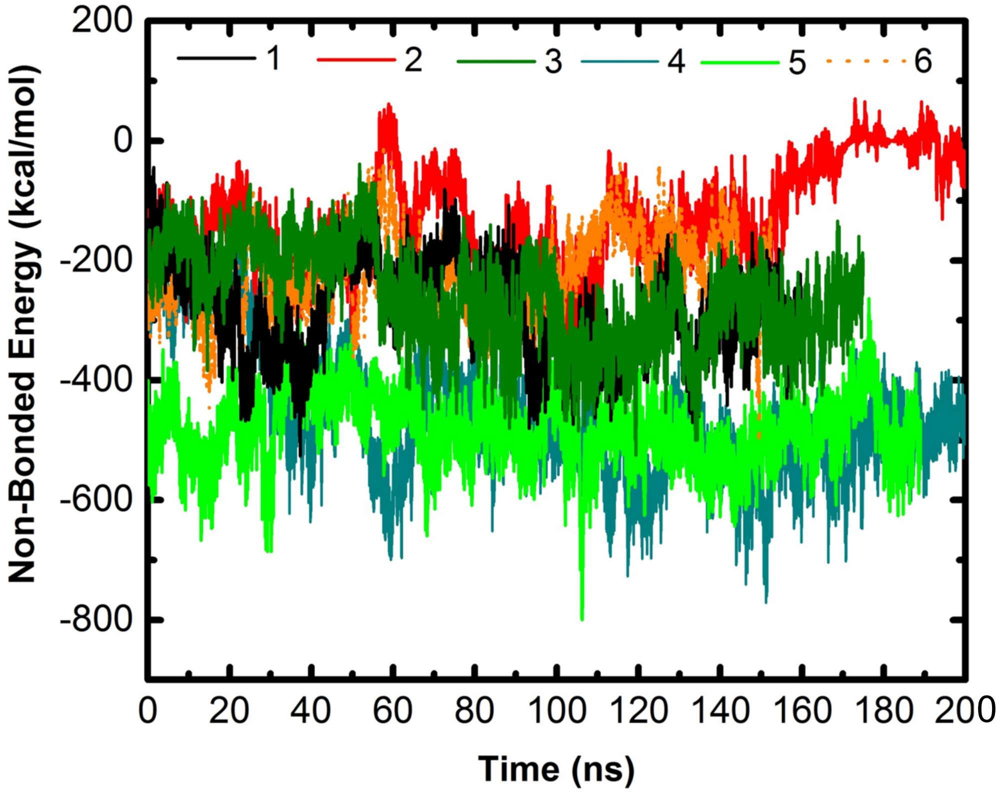


***Figure S3:*** *Time evolution of inter-monomer non-bonded interaction energies during MD simulation of the six predicted models (see table 2 for numbering) in a bilayer. Notice the increase in the energy of two dimers that eventually dissociated (orange and red), and the overall decrease in the rest (stable) models.*

**Computational alanine scanning**

Analyses of interfacial interactions in the MD trajectories identified a number of key residues that stabilize the predicted dimers. Examples are shown in Figure S4, where we plotted the time evolution of the distance between selected inter-monomer hydrogen bonding pairs across the i1 and i2 interfaces. Some interactions present in the beginning remained stable throughout the simulation (e.g., H94-Y137 in i1) while new contacts get formed (e.g. E168-R135 in i2). To complement this analysis, we estimated the relative importance of the interfacial residues for stability through computational alanine scanning with Rosetta [14]. Mutation of a number of i1 and i2 interface residues to alanine led to an increase in the calculated free energy change of binding ($\Delta\Delta G_{bind}$); positive $\Delta\Delta G_{bind}$ indicates destabilization of the interface by the mutation and thereby the importance of the residue for stability of the dimer. For example, in i1, H94A yielded $\Delta\Delta G_{bind} \approx 2$ kcal/mol, indicating that H94 located at the center of h3 contributes favorably to dimerization. Other residues with favorable contribution to stability (defined here as $\Delta\Delta G_{bind} >$ 0.5 kcal/mol) included E91, H95, E98, R102 and D132. A similar analysis identified R135, K128

and E168 as important contributors to stability of i2.

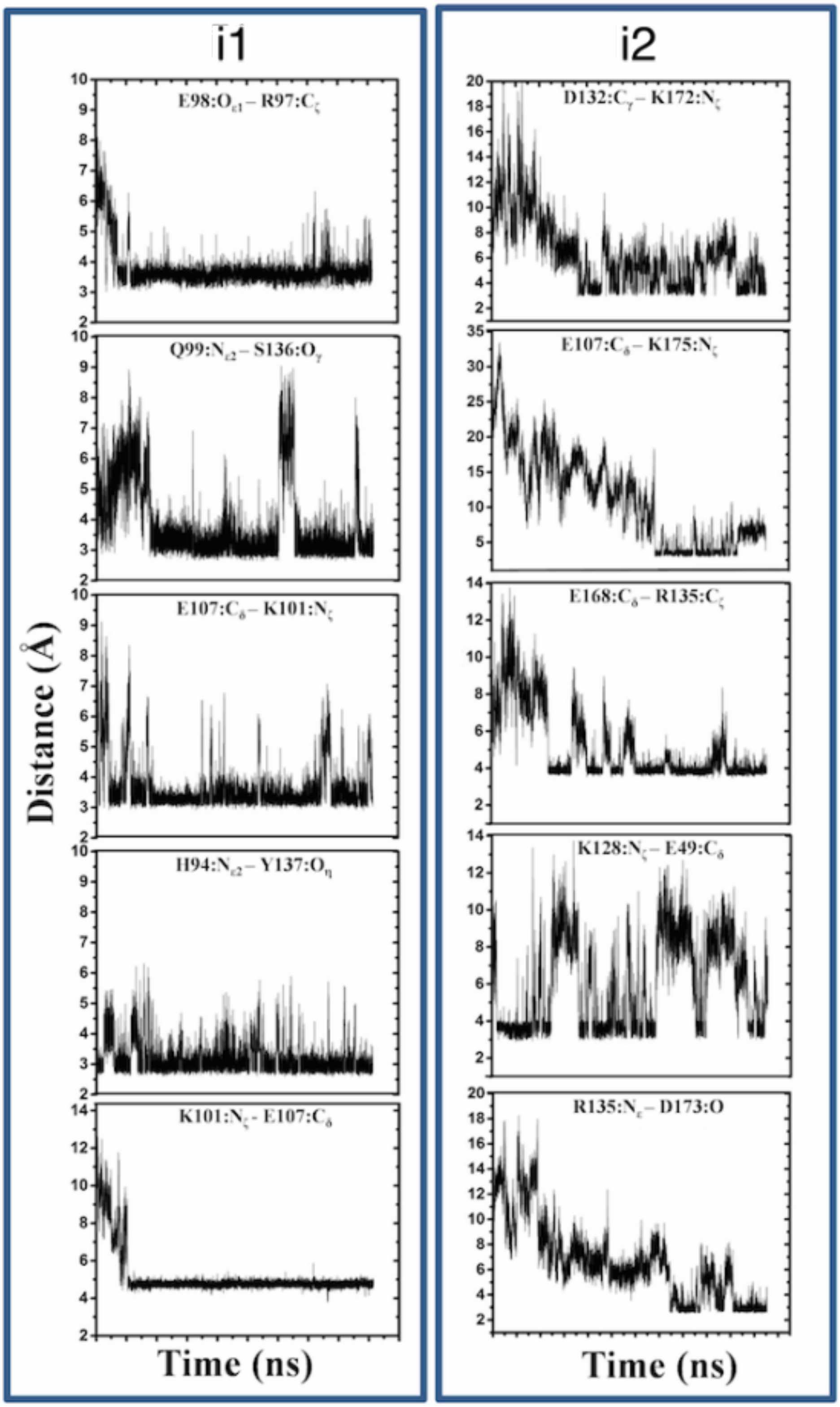


***Figure S4:*** *Time evolution of distances between the indicated atoms of selected residues across the interface of dimer model i1 (left) and i2 (right).*

**Steered molecular dynamics**

We tested the strength of the i1 and i2 interfaces with constant velocity steered molecular dynamics simulations (SMD) [15]. To this end, MD-refined models of i1 and i2 were extracted from membrane and the catalytic domain was re-solvated in a 6.0 x 6.5 x 18.0 $nm^3$ box of water, yielding ~68,000 atom systems after addition of neutralizing ions. These systems were energy minimized and equilibrated as described above. The SMD protocol involved a very slow pulling speed of 5 x $10^{-5}$nm per 0.1fs and a force constant k = 0.1 kcal/mol/$nm^2$. Helices h3/h4 in i1 and h4/h5 in i2 were orientated perpendicular to the z-axis, and the backbone of one protomer was fixed while the center of mass of backbone atoms of the other protomer was pulled along z. The force was recorded every femtosecond, and plotted against time in Figure S5A. We found that dimers required a substantial amount of force to separate, suggesting once again the existence of stable interactions at the interface. Monitoring the progressive rupture of these contacts (Figure S5A & B) provided additional insight into the key stabilizing interactions at the interface.

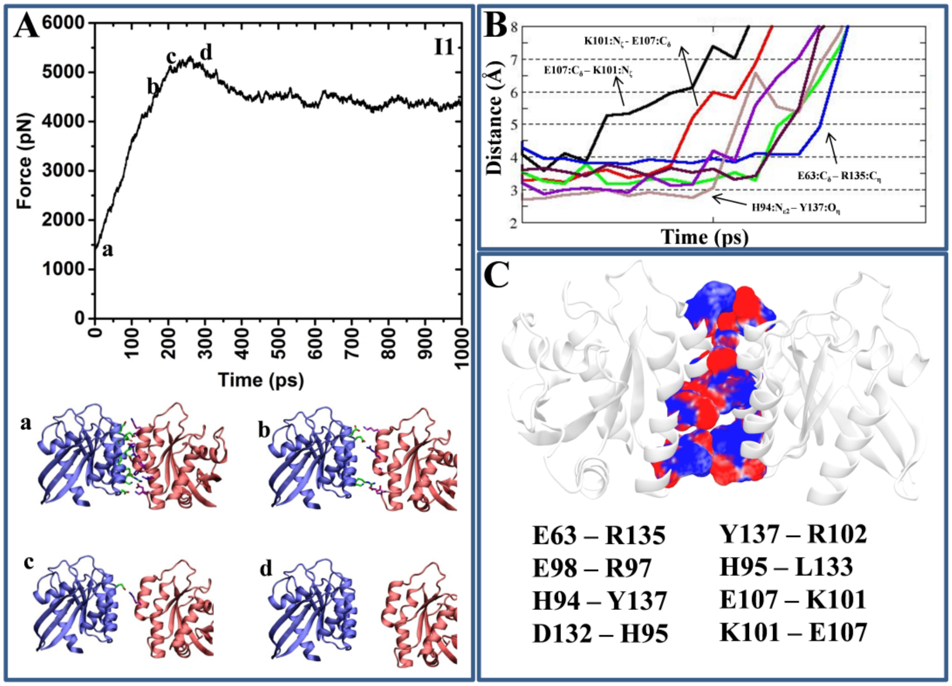


***Figure S5:*** *(A) Force versus time plot (top) from a SMD simulation of i1, showing that a large force is required to separate the monomers. The snapshots (bottom) highlight the extent of separation at the indicated time points of the pulling simulation. (B) Sequential rupture of several inter-monomer interactions (hydrogen bonds) during the dissociation of the dimer. (C) ±2 kT/e electrostatic surface potential calculated with the Adaptive Poisson-Boltzmann Solver on the last snapshot of the MD simulation in bilayer. The calculation was done on the entire dimer but, for clarity, here we show the electrostatic surface potential of only selected residues at the interface. The list of inter-monomer residue pairs at the bottom summarizes the interactions that provide electrostatic stabilization to the dimer as determined by a combined analysis of data from MD, SMD and alanine scanning.*

Combining all of the data from the MD (Table S2 and Figure S3-4), computational alanine scanning, and SMD (Figure S5A & C), we compiled an experimentally testable list of residues that are important for K-Ras dimerization via i1 (Figure S5C) and i2 (E168-R135, E49-K128, K128-E168, D132-K172, and R164-D132). The stability of these interactions was also examined by extended MD in water. Finally, some of these residues were mutated to experimentally evaluate their role in K-Ras dimerization (discussed in the main text).

**The relative orientation of monomers within a dimer**

To estimate fluctuations in the relative orientation of K-Ras monomers during MD simulation of the dimers, we used the azimuthal (θ), polar (ϕ), and Euler angles (α, β, γ) after aligning the principal axis of one monomer (centered at the origin) with the lab axis and defining a vector from the origin to the COM of the other monomer. We then defined an orientation state vector

$$\boldsymbol{q}(t) = \left[\chi_\theta(t) \quad \chi_\phi(t) \quad \chi_\alpha(t) \quad \chi_\beta(t) \quad \chi_\gamma(t)\right] \qquad (1)$$

to characterize the relative orientation of the monomers at time t based on

$$\chi_x(t) = 0.5\left(cos\left(x(t)\right) + 1\right). \qquad (2)$$

Changes in the relative orientation of protomers at different time points can be captured by the cosine of the angle between the corresponding orientation vectors

$$cos(\delta) = \boldsymbol{q}(t) \cdot \boldsymbol{q}(0)/|\boldsymbol{q}(t)||\boldsymbol{q}(0)|). \qquad (3)$$

**Calculation of potential of mean force and equilibrium dissociation constant**

We calculated the PMF of K-Ras dimerization using the Adaptive Biasing Force (ABF) method along the inter-monomer center-mass distance ($d_{COM}$). The setup and equilibration procedures were the same as those described above for simulations in water. During the ABF simulations, the reaction path was divided into overlapping windows of size 0.5-1.2nm using the multiple walker strategy [16]. We chose large windows for better sampling of orthogonal degrees of freedom, and used a harmonic restraint force of 0.5kcal/mol/nm$^2$ to prevent drift outside a window. Each window was sampled for 30-40ns with each walker being 6-7ns long. Additional windows were inserted as needed to increase sampling in certain regions of $d_{COM}$ deemed poorly sampled in a previous step. Instantaneous forces were accumulated into bins of width 0.01nm after discarding the first 10,000 samples where the ABF was inactive. The mean force at the i$^{th}$ global bin, $\langle F\rangle_i$, was calculated as

$$\langle F\rangle_i = \frac{\sum_{j=1}^{N_i} n_{ij}\langle F\rangle_{ij}}{\sum_{j=1}^{N_i} n_{ij}} \qquad (4)$$

where $\langle F \rangle_{ij}$ and $n_{ij}$ are the mean force and number of samples for the $j^{th}$ window at the local bin corresponding to the $i^{th}$ global bin, respectively. $N_i$ is the number of windows that contain the $i^{th}$ global bin and j loops over all such windows. The PMF was obtained by integrating the mean force over $d_{COM}$ using the midpoint rule method, and the mean force in each global bin was smoothed out using a weighted running average of neighboring bins within 0.05nm. $\Delta G_{bind}$ was calculated from the equilibrium dissociation constant ($K_d$) of dimerization estimated from the one-dimensional PMF profile, $u(r)$, as

$$K_d \approx \frac{8\pi^2}{C_0 \Delta\omega \int_{bound} r^2 dr \exp\left(-\frac{u(r)}{RT}\right)} \tag{5}$$

Where $C_0$ and $\Delta\omega$ are, respectively, the standard concentration and the orientational freedom of the bound monomers estimated from the ranges and averages of angular locations and relative orientations extracted from MD trajectories of i1 (~1.43 rad) and i2 (4.99 rad), yielding $K_d$ = 5mM and 107mM for dimers i1 and i2, respectively.

**Electron microscopy and spatial mapping**

Plasma membrane (pm) sheets from baby hamster kidney (BHK) cells transiently expressing mGFP-tagged K-RasG12V mutants were fixed and labeled with anti-GFP antibody conjugated to a 4.5nm gold particle as previously described [17-19]. Digital images of the immunogold-labeled pm sheets were taken in a transmission electron microscope. Intact 1$\mu$m$^2$ areas of the pm sheet were identified using ImageJ, and the *(x,y)* coordinates of the gold particles were determined [18,19]. A univariate K-function was calculated and standardized on the 99% or 95% confidence interval (C.I.). *L(r)-r* greater than the C.I. indicates significant clustering and the maximum value of the function ($L_{max}$) estimates the extent of clustering. Differences between replicated point patterns were analyzed by constructing bootstrap tests as described previously [20,21]; statistical significance evaluated against 1,000 bootstrap samples. Prism (Version 5.0c, GraphPad Software) was used for one-way ANOVA tests.

**Fraction of dimers and higher oligomers from EM spatial analysis**

We calculated the ratios of dimers to monomers and higher oligomers to monomers to examine the impact of the charge reversal and cysteine mutations at i1, which are discussed in the main text, on the distribution of different oligimeric states of K-Ras. Figure S6 shows that the charge reversal point mutations and especially the cysteine double mutant significantly altered the ratios. The single mutants that introduce electrostatic repulsion at the interface reduced the

proportion of dimers and oligomers relative to the monomer fraction. In contrast, the double cysteine mutations that were meant to form an inter-monomer disulfide bridge dramatically increased the fraction of both dimers and higher oligomers. Together, these results show both the link between dimers and higher oligomers and the importance of the computationally derived interfacial residues for multimer formation /stability.

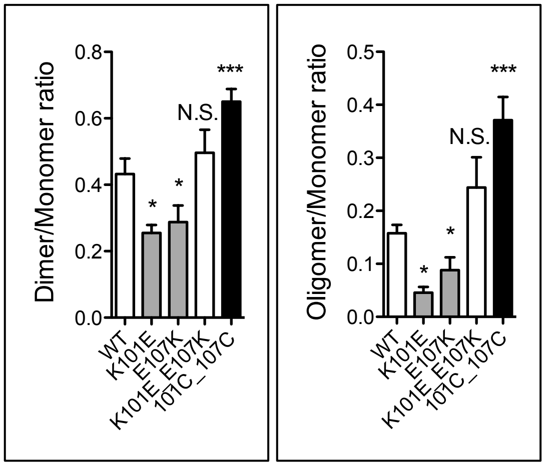


***Figure S6:*** *Ratio of dimers to monomers (left) and higher oligomers (3 or more subunits) to monomers (right) derived from EM analysis of plasma membrane sheets from BHK cells expressing mGFP-K-RasG12V mutants labeled with an anti-GFP conjugated gold particle (for details see Figure 5 in the main text).*

**References**


1. Gorfe, A.A., Grant, B.J. & McCammon, J.A. Mapping the nucleotide and isoform-dependent structural and dynamical features of Ras proteins. *Structure* **16**, 885-96 (2008). doi: 10.1016/j.str.2008.03.009.
2. Grant, B.J., Rodrigues, A.P., ElSawy, K.M., McCammon, J.A. & Caves, L.S. Bio3d: an R package for the comparative analysis of protein structures. *Bioinformatics* **22**, 2695-6 (2006). doi: 10.1093/bioinformatics/btl461
3. Skjaerven, L., Yao, X.Q., Scarabelli, G. & Grant, B.J. Integrating protein structural dynamics and evolutionary analysis with Bio3D. *BMC Bioinformatics* **15**, 399 (2014). doi: 10.1186/s12859-014-0399-6.
4. Gray, J.J. et al. Protein-protein docking with simultaneous optimization of rigid-body displacement and side-chain conformations. *J Mol Biol* **331**, 281-99 (2003). doi:10.1016/S0022-2836(03)00670-3
5. Prakash, P., Zhou, Y., Liang, H., Hancock, J.F. & Gorfe, A.A. Oncogenic K-Ras Binds to an Anionic Membrane in Two Distinct Orientations: A Molecular Dynamics Analysis. *Biophys J* **110**, 1125-38 (2016). doi: 10.1016/j.bpj.2016.01.019.
6. Chaudhury, S. et al. Benchmarking and analysis of protein docking performance in Rosetta v3.2. *PLoS One* **6**, e22477 (2011). doi: 10.1371/journal.pone.0022477.
7. Li, S.C. & Ng, Y.K. Calibur: a tool for clustering large numbers of protein decoys. *BMC Bioinformatics* **11**, 25 (2010). doi: 10.1186/1471-2105-11-25.
8. Andre, I., Strauss, C.E., Kaplan, D.B., Bradley, P. & Baker, D. Emergence of symmetry in

homooligomeric biological assemblies. *Proc Natl Acad Sci U S A* **105**, 16148-52 (2008). doi: 10.1073/pnas.0807576105.

9. Phillips, J.C. et al. Scalable molecular dynamics with NAMD. *Journal of Computational Chemistry* **26**, 1781-1802 (2005). DOI: 10.1002/jcc.20289
10. MacKerell, A.D. et al. All-Atom Empirical Potential for Molecular Modeling and Dynamics Studies of Proteins†. *J Phys Chem B* **102**, 3586-3616 (1998). doi: 10.1021/jp973084f.
11. Klauda, J.B. et al. Update of the CHARMM all-atom additive force field for lipids: validation on six lipid types. *J Phys Chem B* **114**, 7830-43 (2010). doi: 10.1021/jp101759q.
12. Darden, T., York, D. & Pedersen, L. Particle mesh Ewald: An N·log(N) method for Ewald sums in large systems. *J Chem Phys* **98**, 10089-10092 (1993). http://dx.doi.org/10.1063/1.464397
13. Ryckaert, J.P.C., G.; Berendsen, H. J. C. Numerical integration of the Cartesian Equations of Motion of a System with Constraints: Molecular Dynamics of n-Alkanes. *J Comput Phys* **23**, 15 (1977). doi:10.1016/0021-9991(77)90098-5
14. Kortemme, T., Kim, D.E. & Baker, D. Computational alanine scanning of protein-proteininterfaces. *Sci STKE* **2004**, pl2 (2004). DOI: 10.1126/stke.2192004pl2
15. Isralewitz, B., Gao, M. & Schulten, K. Steered molecular dynamics and mechanical functions of proteins. *Curr Opin Struct Biol* **11**, 224-30 (2001). doi:10.1016/S0959-440X(00)00194-9
16. Minoukadeh, K., Chipot, C. & Lelièvre, T. Potential of Mean Force Calculations: A Multiple-Walker Adaptive Biasing Force Approach. *J Chem Theory Comput* **6**, 1008-1017 (2010). DOI: 10.1021/ct900524t
17. Cho, K.J. et al. Staurosporines disrupt phosphatidylserine trafficking and mislocalize Ras proteins. *J Biol Chem* **287**, 43573-84 (2012). doi: 10.1074/jbc.M112.424457.
18. Hancock, J.F. & Prior, I.A. Electron microscopic imaging of Ras signaling domains. *Methods* **37**, 165-72 (2005). doi:10.1016/j.ymeth.2005.05.018
19. Prior, I.A., Muncke, C., Parton, R.G. & Hancock, J.F. Direct visualization of Ras proteins in spatially distinct cell surface microdomains. *J Cell Biol* **160**, 165-70 (2003). doi: 10.1083/jcb.200209091
20. Diggle P. J.; Mateu J.;Clough, H.E. A comparison between parametric and non-parametric approaches to the analysis of replicated spatial point patterns. *Adv in Appl Probab* **32**, 13 (2000).
21. Plowman, S.J., Muncke, C., Parton, R.G. & Hancock, J.F. H-ras, K-ras, and inner plasma membrane raft proteins operate in nanoclusters with differential dependence on the actin cytoskeleton. *Proc Natl Acad Sci U S A* **102**, 15500-5 (2005). DOI: 10.1073/pnas.0504114102
